# Description of the L76V Resistance Protease Mutation in HIV-1 B and “Non-B” Subtypes

**DOI:** 10.1371/journal.pone.0054381

**Published:** 2013-01-18

**Authors:** Charlotte Charpentier, Sidonie Lambert-Niclot, Claudia Alteri, Alexandre Storto, Philippe Flandre, Valentina Svicher, Carlo-Federico Perno, Françoise Brun-Vézinet, Vincent Calvez, Anne-Geneviève Marcelin, Francesca Ceccherini-Silberstein, Diane Descamps

**Affiliations:** 1 Laboratoire de Virologie, Assistance Publique-Hôpitaux de Paris (AP-HP), Groupe Hospitalier Bichat-Claude Bernard, HUPNVS, Université Paris Diderot, Paris 7, PRES Sorbonne Paris Cité, EA4409, Paris, France; 2 AP-HP, Hôpital Pitié-Salpêtrière, Laboratoire de Virologie, UPMC Univ Paris 06, INSERM UMR S943, Paris, France; 3 University of Rome “Tor Vergata”, Department of Experimental Medicine and Surgery, Rome, Italy; 4 INSERM UMR-S 943, University Pierre and Marie Curie Paris 6, Paris, France; Centro de Biología Molecular Severo Ochoa (CSIC-UAM), Spain

## Abstract

**Objective:**

To describe the prevalence of the L76V protease inhibitors resistance-associated mutation (PI-RAM) in relation with patients’ characteristics and protease genotypic background in HIV-1 B- and “non-B”-infected patients.

**Methods:**

Frequency of the L76V mutation between 1998 and 2010 was surveyed in the laboratory database of 3 clinical centers. Major PI-RAMs were identified according to the IAS-USA list. Fisher’s and Wilcoxon tests were used to compare variables.

**Results:**

Among the overall 29,643 sequences analyzed, the prevalence of L76V was 1.50%, while was 5.42% in PI-resistant viruses. Since 2008 the prevalence of L76V was higher in “non-B”-infected than in B-infected patients each year. Median time since diagnosis of HIV-1 infection and median time under antiretroviral-based regimen were both shorter in “non-B”- than in B-infected patients (8 *vs* 11 years, *P*<0.0001; and 7 *vs* 8 years, *P* = 0.004). In addition, “non-B”-infected patients had been pre-exposed to a lower number of PI (2 *vs* 3, *P* = 0.016). The L76V was also associated with a lower number of major PI-RAMs in “non-B” *vs* B samples (3 *vs* 4, *P = *0.0001), and thus it was more frequent found as single major PI-RAM in “non-B” *vs* B subtype (10% *vs* 2%, *P = *0.014).

**Conclusions:**

We showed an impact of viral subtype on the selection of the L76V major PI-RAM with a higher prevalence in “non-B” subtypes observed since 2008. In addition, in “non-B”-infected patients this mutation appeared more rapidly and was associated with less PI-RAM.

## Introduction

The antiretroviral drug class of protease inhibitors (PI) is known to have a high genetic barrier to resistance [1]. The recent large clinical trials assessing the efficacy of boosted PI-containing regimen in antiretroviral-naïve patients showed a very low rate of selection of PI resistance-associated mutations (RAM) in case of virological failure [2], [3]. However, a novel resistance pathway involving the protease mutation L76V was recently described both in antiretroviral-naïve patients [4], [5] and in antiretroviral-experienced patients [6]–[9]. The L76V is a drug resistance mutation associated with resistance to lopinavir, darunavir, fosamprenavir and indinavir [4]–[5], [10]. In addition, the L76V is associated with an *in vitro* hypersusceptibility to saquinavir, atazanavir, and tipranavir [11], [12].

The prevalence of the L76V mutation in PI-resistant viruses was found about 3.3% in two large databases of clinical sequences [5], [10], with no viral subtype sub-analysis. Some studies reported a high prevalence of L76V in “non-B” subtypes, particularly in the CRF02_AG recombinant [4], [8]. Firstly, in the MONARK study, assessing lopinavir monotherapy in antiretroviral-naïve patients, the prevalence of the L76V in case of virological failure was 9.4% in this study and all patients displaying L76V-mutated viruses at failure were infected with CRF02_AG recombinant [4]. In a study assessing genotypic resistance profiles in 57 patients living in Cameroon, all infected with HIV-1 “non-B” subtypes, the prevalence of the L76V was 8.8% [8]. However, few data are available on the impact of the viral subtype on the selection of the L76V mutation.

The aim of the study was to describe the L76V protease mutation in term of prevalence, patients characteristics, and PI RAM clustering with the L76V mutation in the context of HIV-1 B subtype and HIV-1 “non-B“ subtypes.

## Patients and Methods

### Database Analysis

Frequency of the L76V mutation was surveyed in the clinical laboratory database of 2 clinical centers in Paris, France (Pitié-Salpêtrière and Bichat-Claude Bernard Hospitals) and 1 in Rome, Italy (University of Rome “Tor Vergata”). Sequences included in the databases of the 3 centers resulted from all the genotypic resistance tests performed in clinical routine requested by the physician during patients’ follow up between 1998 and 2010. This included as well antiretroviral-naïve as antiretroviral-experienced patients. No significant difference in the nature of ARV-based treatment prescribed was observed according to the center. Similar demographic characteristics were observed among the patients followed in the 3 centers of the study (data not shown), except for the proportion of HIV-“non-B”-infected patients that is lower in the Roman centre (18%) than in the Parisian centers (42% and 51%). In our study, samples with at least one of the major PI RAM of the IAS-USA list as follows: D30N, V32I, M46I/L, I47A/V, G48V, I50L/V, I54L/M, Q58E, T74P, L76V, V82A/F/L/T/S, N83D, I84V, N88S, L90M were considered as PI-resistant issued from PI-experienced patients [13]. In the case of multiple samples from the same patient we only taking into account the first chronological sample harboring the L76V mutation.

### Genotypic Resistance Tests

Population-based sequencing of protease and reverse transcriptase were performed in the 2 Parisian centers using an in-house PCR assay according to the complete sequencing procedures and primers sequences described at www.hivfrenchresistance.org. The Roma center used a commercial assay (ViroSeq® HIV-1 genotyping system, Celera Diagnostics, Alameda, Ca), as previously described [14]. Resistance mutations and major PI RAMs were identified according to the IAS-USA list [13].

### HIV-1 Subtyping

HIV-1 subtype was determined by phylogenetic analyses, by estimating the relationships among RT sequences and reference sequences of HIV-1 genetic subtypes and circulating recombinant forms (CRF) obtained from the Los Alamos Database (http://hiv-web.lanl.gov). Phylogenetic trees were inferred using the neighbour-joining method and two Kimura parameters with 1000 bootstrap values.

### Mutations Covariation Analysis

The association of the L76V mutation with other PI RAM was assessed in a subset of 1,956 subtype B and 481 subtypes “non-B” sequences obtained from patients failing their last PI-based regimen, with a full-length protease sequence available at the time of failure, including sequences without L76V mutation.

To identify significant patterns of pairwise correlations between the L76V mutation and specific PI RAM observed in isolates from PI-experienced patients, we calculated the binomial correlation coefficient (phi) and its statistical significance for each pair of mutations. Average linkage hierarchical agglomerative cluster analysis was performed to investigate if the protease mutations pairwise associated with the L76V mutation raised in specific evolutionary pathways, as previously described [15].

### Statistical Analysis

To compare variables between HIV-1 B- and “non-B”-infected patients the Wilcoxon test and the Fisher exact test were used with a *P*-value threshold >0.002 (Bonferroni correction). All tests were two-sided at the 0.05 significance level. Analyses were performed with StatEL statistical software (StatEL Base, www.adscience.eu).

## Results

### Prevalence of L76V Mutation Over Time

A number of 29,643 sequences issued from clinical samples collected between 1998 and 2010 were available in the database. Among them, 24,604 sequences are issued from antiretroviral-treated patients (83%) and 19,861 sequences are HIV subtype B (67%). Among the 29,643 sequences, 446 displayed the L76V mutation in protease, leading to an overall prevalence of 1.50%. 138 out of the 446 L76V-mutated sequences are HIV-1 subtype “non-B” (31%).

When regarding the prevalence of the L76V mutation among the PI-resistant viruses, containing at least one major PI RAM, it was found at 5.42% (430/7,934).

**Figure 1:**
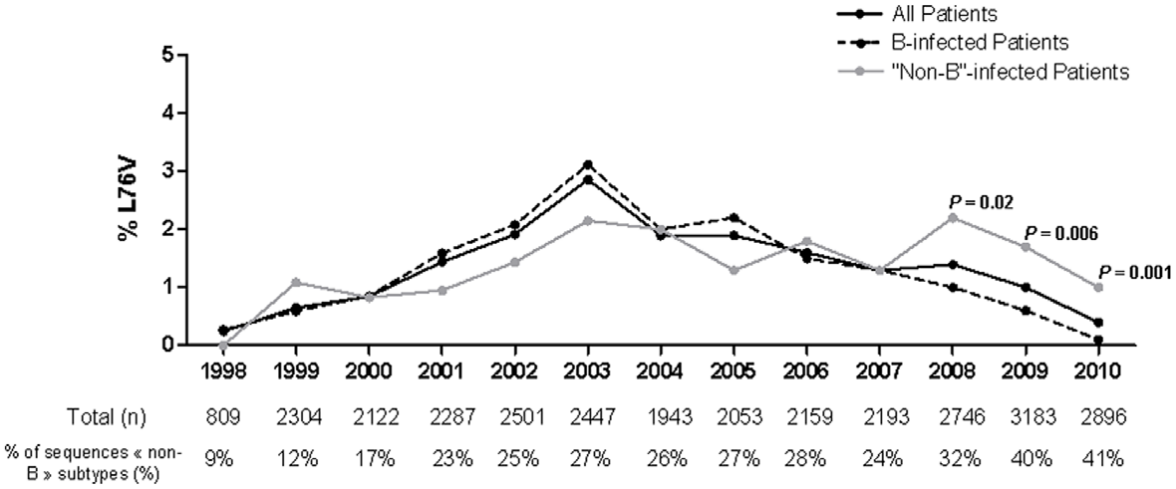
Prevalence of the L76V mutation between 1998 and 2010 in a database containing 29,643 sequences issued from clinical samples with any antiretroviral drug resistance.

In our database, a similar prevalence of the L76V mutation was observed among B- and “non-B”-infected patients until 2008 (Figure 1). Since 2008 the prevalence of L76V was higher in “non-B”-infected than in B-infected patients each year (*P* = 0.02 in 2008, *P* = 0.006 in 2009; and *P* = 0.001 in 2010). In our database the proportion of “non-B” subtypes increased from 27% to 41% between 2003 and 2010.

### Demographic, Therapeutic, and Virological Characteristics of Patients Displaying L76V-mutated Viruses

Regarding the 446 samples with L76V mutation in PI-experienced patients, known clinical and therapeutic history for further analyzes were available in 179 patients. Among them 118 (66%) were infected with HIV-1 subtype B and 61 (34%) with HIV-1 “non-B“ subtypes. The CRF02_AG recombinant form was the most prevalent “non-B” subtype found in 29 samples (47%). The distribution of the remaining HIV-1 “non-B” subtypes was as follows: subtype A (*n* = 6, 10%), D (*n* = 6, 10%), F (*n* = 4, 6%), G (*n* = 4, 6%), CRF06_cpx (*n* = 2, 3%), CRF11_cpx (*n* = 1, 2%), CRF12_BF (*n* = 1, 2%), C (*n* = 1, 2%), H (*n* = 1, 2%), and 6 samples with undetermined subtype.

At time of the first detection of the L76V mutation, median time since HIV infection diagnosis and median time under antiretroviral-based regimen were both shorter in “non-B” patients compared with subtype B patients (8 *vs* 11 years, *P*<0.0001; and 7 *vs* 8 years, *P* = 0.004, respectively) (Table 1). Similar results were obtained if we compared patients infected with HIV-1 subtype B samples to those infected with the CRF02_AG recombinant (data not shown).

**Table 1:** Population characteristics of patients infected with HIV-1 subtype B and HIV-1 “non-B” subtypes with L76V protease mutation.

Populations parameter*	Patients infected with HIV-1 subtype B (*n* = 118)	Patients infected with HIV-1 subtype “non-B” (*n* = 61)	*P*-value
Age (years)	42 (37–48)	41 (36–48)	0.2
Gender n(%) women	16 (14)	29 (48)	**0.00001**
Time since HIV infection diagnosis (years)	11 (8–15)	8 (6–11)	**0.00001**
Time since initial ARV-based regimen (years)	8 (6–11)	7 (4–9)	**0.004**
HIV-1 RNA level (log_10_ copies/mL)	4.31 (3.63–5.12)	4.13 (3.27–4.94)	0.71
PI received at time of the L76V initial detection n(%)			
lopinavir	73 (62)	34 (56)	0.39
indinavir	14 (12)	10 (16)	0.41
amprenavir/fosamprenavir	18 (15)	5 (8)	0.17
saquinavir	8 (7)	7 (11)	0.29
darunavir	8 (7)	5 (8)	0.77
nelfinavir	4 (3)	0 (0)	0.30
atazanavir	0 (0)	0 (0)	1.00
tipranavir	0 (0)	0 (0)	1.00
Number of PI received during therapeutic history	3 (2–4)	2 (1–3)	**0.016**
Duration of PI-based regimens during therapeutic history (months)	61 (43–83)	59 (33–81)	0.053
Dual PI regimen n(%)	23 (19)	15 (25)	0.36
PI previously received during therapeutic history n(%)			
lopinavir	90 (76)	46 (75)	0.82
indinavir	97 (82)	38 (62)	**0.041**
amprenavir/fosamprenavir	40 (34)	17 (28)	0.39
saquinavir	54 (46)	19 (31)	0.061
darunavir	7 (6)	5 (8)	0.55
nelfinavir	49 (41)	19 (31)	0.16
atazanavir	5 (4)	0 (0)	0.17
tipranavir	3 (3)	3 (5)	0.41

* Continuous variables were expressed as median and interquartile range (IQR) and categorical variables were expressed as numbers and percentages.

95% CI: 95% Confidence Interval; ARV: antiretroviral; PI: protease inhibitor.

At the time of first detection of the L76V mutation, there was no significant difference in the nature of PI received between patients infected with B and “non-B” subtypes (Table 1). However, patients infected with B subtype received more frequently lopinavir than patients infected with CRF02_AG (62 *vs* 41%, *P* = 0.04).

We also assessed the PI pre-exposure showing that patients infected with “non-B” subtypes received a lower number of PI before the selection of the L76V mutation than those infected with B subtype (2 *vs* 3, *P* = 0.016), although the duration of PI-based regimen was similar in the 2 groups of patients. There was no difference in the nature of the PI previously received except for indinavir, more frequently received by subtype B patients than by “non-B” patients (82 *vs* 62%, *P* = 0.041). Similar results were obtained if we compared patients infected with HIV-1 subtype B samples to those infected with the CRF02_AG recombinant.

Among the 179 patients of the study, 41 (23%) displayed plasma virus with L76V, although they never received lopinavir. Most of these patients (*n* = 38, 93%) received indinavir in their therapeutic history. There was no difference in the proportion, in the demographic characteristics, and in the pre-therapeutic history of these patients depending of the viral subtype.

### Protease Mutation Patterns of L76V-mutated Viruses among HIV-1 B and “non-B” Subtypes

The number of major PI RAMs associated with L76V mutation was lower in “non-B” samples than in subtype B samples (3 *vs* 4, *P* = 0.0001). Only 8 samples (8/179, 4.5%) were found with L76V as the sole PI RAM, with a higher frequency in “non-B” than in subtype B samples (10% *vs* 2%, *P* = 0.014). Among the 8 patients exhibiting plasma virus with the single L76V mutation, 5 were receiving a lopinavir-based therapy as first line regimen, and half were infected with CRF02_AG (*n* = 4).

**Figure 2:**
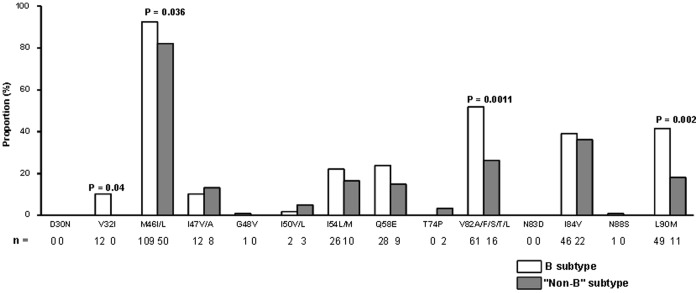
Proportion of protease inhibitors resistance-associated mutations with the L76V mutation in HIV-1 subtype B samples and in HIV-1 “non-B” samples. P-values are indicated only if significant.

The prevalence of major PI RAMs found with the L76V is depicted in Figure 2. The most prevalent PI RAM detected with L76V was the M46I/L, both in subtype B and “non-B” samples, found in 92 and 82% of cases, respectively. Then, the most prevalent mutation found with L76V was the V82A/F/L/T/S (52%) in subtype B samples and I84V (36%) in “non-B” samples. Significant differences in the prevalence of PI RAMs detected with the L76V between subtype B and “non-B” samples were found at 4 positions with a higher prevalence of the V32I, M46I/L, V82A/F/L/T/S and L90M mutations in subtype B than in “non-B” samples: 10 *vs* 0%, *P* = 0.04; 92 *vs* 82%, *P* = 0.036; 52 *vs* 26%, *P* = 0.0011; and 42 *vs* 18%, *P* = 0.002, respectively.

### Analysis of Covariation of L76V among Protease Mutations

Among subtype B samples, the major PI RAM significantly correlating as pairs with L76V were: M46I, I54L/M, Q58E, V82F, I84V, and L90M. The strongest associations were observed with the M46I (covariation frequency 25.9%, phi = 0.39), and I84V major PI RAM (covariation frequency 28.4%, phi = 0.28). The L90M mutation was co-present with the L76V in 49 (11.6%) patients (phi = 0.12). Regarding secondary PI RAM the strongest associations were found with the mutations K55R (covariation frequency 36.4%, phi = 0.32), I54V (covariation frequency 23.3%, phi = 0.27), and L33F (covariation frequency 27.2%, phi = 0.27). Similar pairwise correlations were observed in “non-B” sequences (phi >0.10, *P*<0.0001), with an additional correlation with the I47V major PI RAM (covariation frequency 72.8%, phi = 0.27).

**Figure 3:**
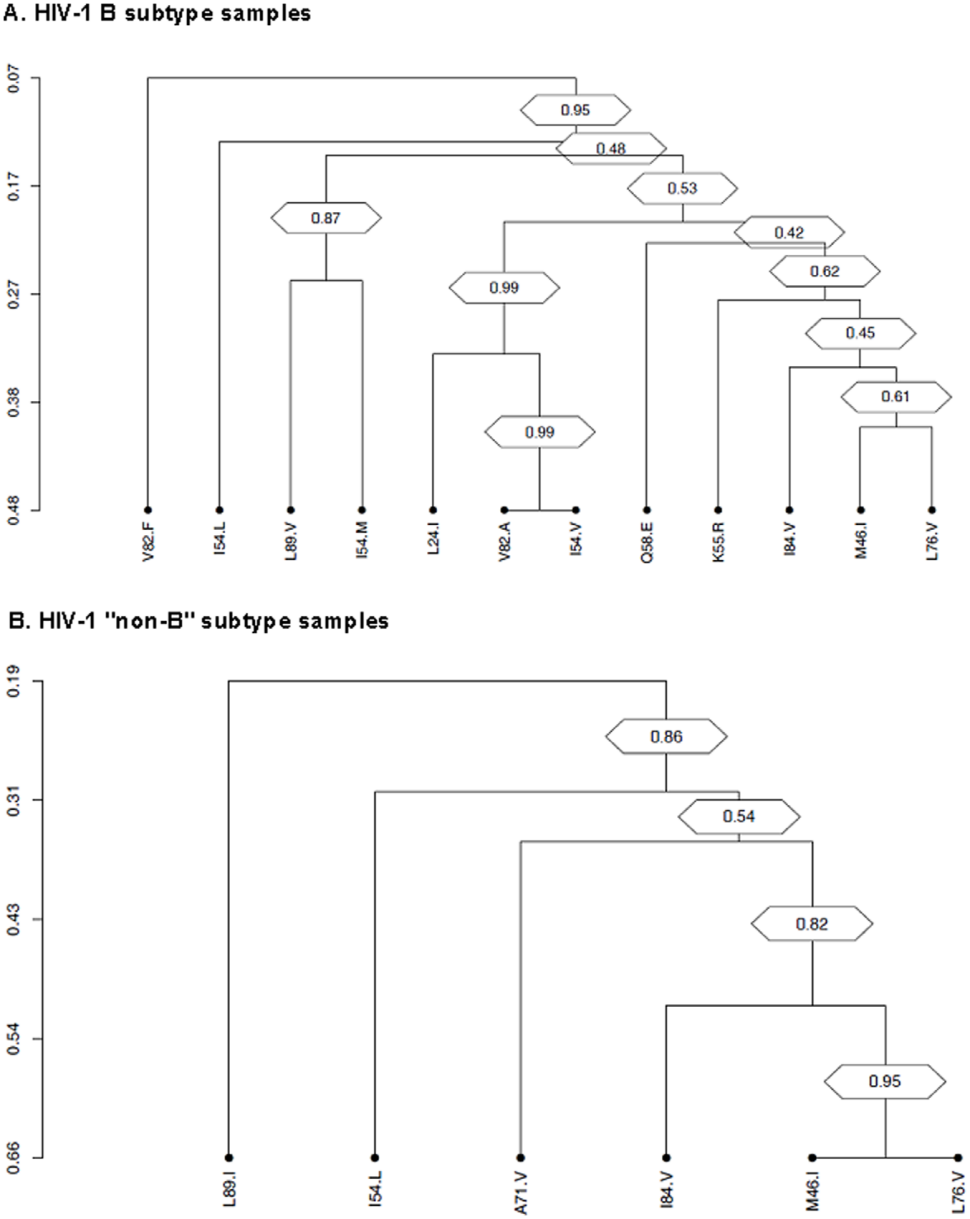
Dendrograms obtained from average linkage hierarchical agglomerative clustering, showing significant clusters of L76V protease inhibitors resistance mutations among B subtype sequences (A), and among “non-B” subtype sequences (B). The length of branches reflects distances between mutations in the original distance matrix. Bootstrap values, indicating the significance of clusters, are reported in the boxes.

Furthermore, we performed average linkage hierarchical agglomerative cluster analysis to investigate if the protease mutations pairwise associated with L76V raised in specific evolutionary pathways. In subtype B samples, the strongly correlated pairs of mutations L76V and M46I clustered along with the I84V and K55R mutations. This cluster was linked to L24I, I54V, and V82A mutations. As a whole, this cluster was highly significant (bootstrap value = 0.78) (Figure 3A).

Likewise, this cluster was confirmed also in HIV-1 “non-B” sequences, where the major PI RAM L76V, M46I, I54L and I84V grouped together with the secondary one A71V (bootstrap value = 0.86). Again, the topology of the dendrogram showed the strong association between L76V and M46I (bootstrap value = 0.95) (Figure 3B).

### Virological Response to the Subsequent Antiretroviral-based Regimen

Virological response to the subsequent antiretroviral-based regimen was assessable in 108 patients of the study at month 3 (M3) and in 98 patients at month 6 (M6). Overall, 20% and 28% of patients displayed HIV-1 RNA below 50 copies/mL at M3 and M6, respectively. The highest rate of virological success was observed with darunavir-based regimen showing 50% (*n* = 13) of patients in success at M3 and 64% (*n* = 14) at M6. At M6, a higher rate of virological success was observed in patients infected with HIV-1 “non-B” than in those infected with B subtype (50% *vs* 26%, *P* = 0.03).

## Discussion

In the present study based on 179 patients, including 61 “non-B”-infected patients, exhibiting plasma virus with the L76V major drug resistance mutation in the protease region we showed that the selection of this mutation appeared earlier in infection history, earlier in therapeutic history and occurred in virus harboring a lower number of major PI resistance mutations in “non-B”-infected patients compared to subtype B-infected patients.

Some limitations of our study might be that “non-B” subtypes constitute a heterogeneous group with low number of sequences available for each subtype preventing subtypes specific analyzes except for CRF02_AG.

Overall in our database containing more than 29,000 sequences, we described a prevalence of the L76V mutation of 1.50% in all sequences and of 3.94% in viruses issued from PI-experienced patients. This prevalence is similar to that described in previous studies reporting 1.16% of L76V in the study of Nijhuis et al. in all viruses and 3.4% and 3.2% in the studies of Nijhuis et al. and Norton et al., respectively, in PI-resistant viruses. In our study, the overall prevalence of L76V tends to decrease during the study period, with no statistical significance. The overtime decrease of prevalence has been recently also described for the M184I/V and K103 resistance mutations [16]. The more effective and better tolerated antiretroviral-based regimens likely contribute to the decreased population trends of drug resistance.

In the present study we described that the prevalence of L76V since 2008 is significantly higher in “non-B”-infected patients than in subtype B-infected patients. To our knowledge, this is the first time that a differential prevalence of the L76V depending of the viral subtype was described.

In our study a shorter time since HIV diagnosis, a shorter time under antiretroviral-based therapy, and a lower number of PI in the therapeutic history were all significantly observed in ”non-B”-infected patients when compared to B-infected patients exhibiting L76V-mutated viruses. In the study of Champenois et al. demographic and clinical data were similar between patients harboring L76V-mutated viruses and those with wild-type residue at position 76, no viral subtype analysis was performed [17].

We also showed that the L76V mutation was associated with a lower number of major PI RAMs in “non-B” than in subtype B sequences. We did not analyze minor PI RAMs, as these positions might be polymorphic in “non-B” subtypes. Moreover, no reference lists of mutations are available for “non-B” subtypes. The most prevalent PI RAM detected with L76V was the M46I mutation, whatever the viral subtype, may be due to the use of indinavir. Interestingly, a higher prevalence of virus exhibiting the L76V mutation as single major PI RAM was observed in “non-B” sequences than in B sequences (10% *vs* 2%, respectively). In the database assessed by Young et al., the prevalence of L76V as single PI RAM was rare, found in 0.04% of the samples [10].

Several hypotheses may explain this apparent easier selection of the L76V mutation in the context of “non-B” subtypes. Firstly, the genetic barrier, defined as the number of viral mutations required to overcome the drug-selective pressure, is one of the important factors in the development of drug resistance. Differences have been previously observed in the genetic barrier to resistance in mutations associated with resistance to non nucleoside reverse transcriptase inhibitors (NNRTI) for the A98S and V106M mutations between HIV-1 B and C subtypes [18], [19], or in mutations associated with resistance to integrase inhibitors between B and CRF02_AG subtypes [20]. When regarding the position 76 of the protease at the nucleotidic level, we observed a high degree of conservation suggesting a similar genetic barrier between B and CRF02_AG sequences (data not shown). These findings suggest that there is no lower genetic barrier to acquire the L76V in CRF02_AG than in subtype B, with a change from T to G in both cases.

Secondly, HIV-1 “non-B” subtypes protease sequences exhibit several natural polymorphisms, and some are associated with resistance PI drug class. This specific genotypic background of “non-B” subtypes protease may have a possible role in a more rapid selection of the L76V mutation. The covariation analysis we performed in our study showed that the L76V mutation clustered with the major PI RAM M46I as described in previous studies both in B and non-B viruses [4]–[5], [8]. In our study, the covariation analysis did not allow to evidence different clustering of the L76V PI RAM with protease polymorphisms or other PI RAM according to the viral subtype. These findings suggest that the more rapid selection of the L76V observed in “non-B” subtypes might not be explained by an association of the L76V with a specific protease polymorphism or PI RAM in “non-B” subtype.

Previous studies demonstrated the role of *gag* cleavage sites mutations in the development of resistance to PI [21]–[23]. In the MONARK study, assessing the PI monotherapy strategy in antiretroviral-naïve patients, “non-B” subtypes isolates were significantly more likely to harbor mutations in *gag* cleavage sites at baseline than B subtype isolates (*P*<0.0001) [24]. In our study, one hypothesis might be that the presence of pre-therapeutic *gag* polymorphisms could favor the selection of the L76V mutation. This hypothesis needs further investigation.

Regarding the virological response to subsequent regimen, the highest rate of virological success (64%) was observed with darunavir-based regimen. In our study we showed a higher rate of virological success in the subsequent regimen in patients infected with HIV-1 “non-B” than in those infected with B subtype, we can make the hypothesis that it could be related to the lower number of PI RAMs observed in “non-B” infected patients. However, this part of the study have several limitations, as the limited number of patients and the fact that the study reports on different periods during which optimal antiretroviral-based regimens were not always used.

In conclusion, in this study assessing the prevalence of L76V PI RAM in “non-B”- and B-infected patients, we showed that the viral subtype could have an impact on the selection of this mutation. For the first time a higher prevalence of the L76V mutation in “non-B”- than in B-infected patients was observed since 2008. In addition, the L76V mutation appeared more rapidly and was associated with less PI-RAM in “non-B” subtypes than in B subtype. Further structural and/or *in vitro* experiments are needed to better explain this phenomenon. In addition, further studies based on immuno-virological outcome of HIV-1 “non-B”-infected patients receiving PI-based regimen, especially in resource-limited settings, might help to assess the clinical implications of the presence of the L76V mutation.
